# “A Question of Trust” and “a Leap of Faith”—Study Participants’ Perspectives on Consent, Privacy, and Trust in Smart Home Research: Qualitative Study

**DOI:** 10.2196/25227

**Published:** 2021-11-26

**Authors:** Mari-Rose Kennedy, Richard Huxtable, Giles Birchley, Jonathan Ives, Ian Craddock

**Affiliations:** 1 Centre for Ethics in Medicine University of Bristol Bristol United Kingdom; 2 Department of Electrical & Electronic Engineering University of Bristol Bristol United Kingdom

**Keywords:** smart homes, assistive technology, research ethics, informed consent, privacy, anonymization, trust

## Abstract

**Background:**

*Ubiquitous*, *smart* technology has the potential to assist humans in numerous ways, including with health and social care. COVID-19 has notably hastened the move to remotely delivering many health services. A variety of stakeholders are involved in the process of developing technology. Where stakeholders are research participants, this poses practical and ethical challenges, particularly if the research is conducted in people’s homes. Researchers must observe prima facie ethical obligations linked to participants’ interests in having their autonomy and privacy respected.

**Objective:**

This study aims to explore the ethical considerations around consent, privacy, anonymization, and data sharing with participants involved in SPHERE (Sensor Platform for Healthcare in a Residential Environment), a project for developing smart technology for monitoring health behaviors at home. Participants’ unique insights from being part of this unusual experiment offer valuable perspectives on how to properly approach informed consent for similar smart home research in the future.

**Methods:**

Semistructured qualitative interviews were conducted with 7 households (16 individual participants) recruited from SPHERE. Purposive sampling was used to invite participants from a range of household types and ages. Interviews were conducted in participants’ homes or on-site at the University of Bristol. Interviews were digitally recorded, transcribed verbatim, and analyzed using an inductive thematic approach.

**Results:**

Four themes were identified—motivation for participating; transparency, understanding, and consent; privacy, anonymity, and data use; and trust in research. Motivations to participate in SPHERE stemmed from an altruistic desire to support research directed toward the public good. Participants were satisfied with the consent process despite reporting some difficulties—recalling and understanding the information received, the timing and amount of information provision, and sometimes finding the information to be abstract. Participants were satisfied that privacy was assured and judged that the goals of the research compensated for threats to privacy. Participants trusted SPHERE. The factors that were relevant to developing and maintaining this trust were the trustworthiness of the research team, the provision of necessary information, participants’ control over their participation, and positive prior experiences of research involvement.

**Conclusions:**

This study offers valuable insights into the perspectives of participants in smart home research on important ethical considerations around consent and privacy. The findings may have practical implications for future research regarding the types of information researchers should convey, the extent to which anonymity can be assured, and the long-term duty of care owed to the participants who place trust in researchers not only on the basis of this information but also because of their institutional affiliation. This study highlights important ethical implications. Although autonomy matters, trust appears to matter the most. Therefore, researchers should be alert to the need to foster and maintain trust, particularly as failing to do so might have deleterious effects on future research.

## Introduction

### Background

Recent technological advances have made it possible to embed computer devices in everyday environments and objects [1,2]. *Ubiquitous*, *smart*, and assistive computing technology such as sensors, cameras, and interfaces, which can wirelessly connect and communicate, can aid humans in numerous ways [1,2]. For example, such technology has the potential to improve health and health care by monitoring medical conditions and providing in-home assistance [3-5]. The advent of COVID-19 has notably hastened the move to remote delivery of many health services, such as primary care [6].

Before technology can deliver on its promise, robust research is needed, which (in part) requires attention to the needs and perceptions of intended users. Research in this area rightly tends to be participatory, with potential users involved in study development and evaluation [5,7]. However, conducting research on ubiquitous technologies can pose both practical and ethical challenges, particularly if the study situates technology in real homes [8]. Participants should not expect to benefit directly from such interventions [9], and even if some benefit might accrue, the participants might be drawn from vulnerable populations and have complex needs [7].

Whoever the research participant is, they have interests in their autonomy and privacy being respected wherever and whenever they might contribute to the study. Therefore, there are prima facie ethical obligations to observe. First, participation must be *consensual*. Respect for autonomy requires the provision of consent, which is voluntarily given by a (mentally) competent individual who is sufficiently well informed. However, consent can be challenging, particularly in this context. Some participants, such as young children in the household or those with dementia, whom such research *might* come to benefit, may have absent or diminished competence to consent. Information about the study (such as what the technology does, the extent to which pseudonymization offers privacy, or the security of the technology) can also be difficult to grasp, even by those participants whose competence is unimpaired. Full disclosure of information to participants will not always be possible in any event, given potential unanticipated uses to which the data gathered might be put in the future; in this case, broad consent for the secondary uses of data is required. Researchers need to be attentive to such challenges and to the means of overcoming them, for example, by ensuring that consent is a process rather than an event and being open about the uncertain uses data gathered with broad consent may be put to in the future [10].

Second, some claim that researchers should also be mindful of the need to respect *privacy* [11,12]. Concerns about privacy might be especially acute when equipment is installed in and data gathered from people’s homes [8,13]. However, there is debate about whether privacy is a concern in itself or whether privacy concerns can be met by attention to other protections, so responding to privacy concerns is not straightforward [14]. Furthermore, different people rate the importance of privacy differently for a myriad of reasons across different circumstances: what one person considers a problematic invasion of privacy, another might not [12] (eg, differing attitudes toward sharing data with social media platforms [15]). Difficulties in gaining consent and the contested nature of privacy make it clear that, when collecting potentially sensitive personal data, there is a need for researchers to take care in ensuring that arrangements to protect participants’ interests are as effective and appropriate as possible.

### Objectives

Informed by these background ethical considerations, we sought participants’ views on consent and privacy in the context of smart home research. Our participants were drawn from households involved in the SPHERE (Sensor Platform for Healthcare in a Residential Environment) project. SPHERE is an ambitious project led by Professor Ian Craddock at the University of Bristol, involving collaborators from other universities; local third sector and community collaborators, such as Bristol City Council and Knowle West Media Centre; and international partners from the industry (IBM and Toshiba) [16]. The aim of SPHERE is “to develop a multi-purpose, multimodal sensor platform for monitoring people’s health inside their homes” [9]. SPHERE, as described herein, is exploratory in its approach and not directed toward the specific needs of particular user groups. The participants encompass a variety of households, such as couples or families recruited with no particular focus on health conditions; however, subsequent SPHERE studies (outside the scope of the present contribution) specifically include patients with cardiovascular conditions, dementia, and Parkinson disease and those recovering from orthopedic surgery. The team has developed multiple technologies, including hardware, software, and machine learning, and more specifically an in-house Internet of Things platform, comprising environmental, video, and wearable sensors. The focus of this paper is to describe the SPHERE-CARED (Consent and Anonymization: A Review of Ethical Dimensions) study, exploring SPHERE participants’ perspectives on the ethical aspects of informed consent, anonymity, privacy, and data sharing. For a list of the SPHERE sensor technologies, including wearables, ambient sensors, and cameras, the interested reader should refer to the literature [8,17]. SPHERE aims to test the technology, primarily by obtaining data about human movement and ambient measures that could in the future result in home health monitoring applications or the ability to address health research questions over long periods in the patient’s own home, including measuring the characteristics of health-related activities of daily living, such as sleep, cooking, walking, moving between rooms, and transitions from sitting to standing. Prototypes are first rigorously tested for robustness and stability before the sensors are deployed in participants’ homes [9].

The SPHERE system was designed in accordance with the principles of privacy by design [18], including those of data minimization, end-to-end security, respect for user privacy, and the use of data protection impact assessments within the project. Many SPHERE design decisions were arrived at through workshops with members of the public during the system design process.

Before (and during) deployment, SPHERE has sought the views of the public at large via engagement events and involvement in its *Friends of SPHERE* group. Research participants, which include households comprising entire cohabiting families, must consent to participation and are assured that the data gathered are anonymized. Beyond contributing to the generation of knowledge that might prove beneficial in the future, participants do not receive any direct benefit from participation (however, they are recompensed for increased domestic electricity costs arising from their participation).

Participation in SPHERE does not intrude on participants’ bodily integrity (meaning that participation does not require the use of, or intrusion into, participants’ bodies), and careful attention has been paid to protecting the identities of participants. However, the project does involve monitoring, with not only environmental sensors but also cameras, in their homes. Moreover, many participants lived with this level of monitoring in their houses for months or even years, which may be regarded as a considerable invasion of their privacy. Therefore, SPHERE participants are uniquely well-positioned to contribute to research exploring ethical questions regarding surveillance, privacy and confidentiality, and the provision of informed consent in smart home research. The SPHERE-CARED project comprises an empirical study whose objectives are to qualitatively explore SPHERE participants’ views about these ethical issues and use those data to inform reflections on the ethical dimensions of smart home research to benefit and enlighten similar future research. The study was guided by the following two research questions:

1. How do participants of SPHERE understand and think about the ethical issues arising from the SPHERE around informed consent, anonymity, privacy, and data sharing?
2. How can the insights from SPHERE participants about their consent experience help inform general thinking about how informed consent should be approached in future smart home research?

## Methods

### Sampling and Recruitment

SPHERE-CARED participants were recruited from the SPHERE cohort as whole households. Households that were currently participating or had previously participated in SPHERE were eligible for inclusion. SPHERE households recruited via the National Health Service and those who did not consent to follow-up research were excluded. As a result, of the total 50 (95 participants) SPHERE households, 26 (52%; participants: 53/95, 56%) were eligible for SPHERE-CARED. Eligible households were then purposively sampled to obtain the maximum variation [19] within the bounds of quite a limited subset of households. The final sample included participants from a range of age groups and from the following four household types: individuals, families, couples, and shared residences.

Recruitment was conducted in 3 stages. First, the SPHERE deployment officer identified eligible households and made initial contact with them via their preferred method of communication. Second, details of interested households were passed to the SPHERE-CARED researcher (MK), who arranged a telephone call to further explain the project. Finally, interviews were organized with households once it was confirmed that all members were willing to participate. In the first stage, 28% (14/50) of households (participants: 31/95, 33%) were contacted. Of these 14 households, 7 (50%) households (participants: 16/31, 52%) expressed interest in the research and were subsequently contacted by MK. All 7 households were recruited. All 4 household types were represented, and participants’ ages ranged across 9 decades (Tables 1 and 2). The ages are presented as a range to preserve participant anonymity.

**Table 1:** Household demographics (households: n=7; participants: n=16).

Household ID	Household type	Total occupants, n (%)	Association with university	Attendance of >1 pre-event
H01	Family	4 (25)	Yes	Yes
H02	Family	4 (25)	Yes	Yes
H03	Family	2 (13)	Yes	No
H04	Couple	2 (13)	Yes	Yes
H05	Individual	1 (6)	No	Yes
H06	Individual	1 (6)	No	Yes
H07	Shared residence	2 (13)	Yes	No

**Table 2:** Participant demographics (households: n=7; participants: n=16).

Household ID and participant ID			Gender		Age (years), range
**H01**					
	P01	Male		51-60	
	P02	Female		51-60	
	P03	Female		11-16	
	P04	Female		11-16	
**H02**					
	P05	Male		31-40	
	P06	Female		31-40	
	P07	Male		0-5	
	P08	Male		0-5	
**H03**					
	P09	Female		41-50	
	P10	Female		0-5	
**H04**					
	P11	Female		41-50	
	P12	Male		41-50	
**H05**					
	P13	Male		81-90	
**H06**					
	P14	Female		61-70	
**H07**					
	P15	Female		31-40	
	P16	Female		31-40	

### Ethics

SPHERE-CARED was reviewed and granted a favorable opinion by the University of Bristol Faculty of Social Science and Law research ethics committee as an amendment to the original SPHERE project, which was reviewed and granted a favorable opinion by the University of Bristol Faculty of Engineering research ethics committee (reference: FREC40403). In accordance with university policies, the study also required the completion of 2 risk assessments on working with children and lone-working, and a lone-worker protocol was implemented.

As these were household interviews, children were included as participants. Signed informed consent was sought from all participants aged ≥16 years; informed (age-appropriate) verbal assent was sought from younger children, with written informed consent for their inclusion also provided by parents. Adults and children were informed of their right to revoke consent or assent at any time during the interview. In interviews that included younger children, the interviewer (MK) was vigilant for any signs of distress, which would require a review of the assent with the household. All households were provided with a £20 (US $27.2) shopping voucher to thank them for their involvement in this research.

### Data Collection

Qualitative interviews were conducted with 7 households; of the 7 interviews, 6 (86%) were conducted in participants’ homes, and 1 (14%) was conducted on-site at the university in an adapted house equipped with the study smart home technology to aid participants’ recollections of living with ubiquitous technologies. A topic guide was developed to explore participants’ involvement in and experiences of the main SPHERE project. Questions were designed to focus on participants’ views regarding information provision and informed consent, anonymity, and data use, including probing for areas where they may have experienced problems, such as misunderstandings, requirements for further information, or any disagreements among the household (Textbox 1). Interviews were conducted with the whole household in one instance and were semistructured, allowing the researcher (MK) flexibility to follow up on issues raised by participants during the interviews. To facilitate a more comfortable interview environment for younger children, age-appropriate materials were provided; for example, a soft toy was provided to children aged between 0 and 5 years [20]. Children were invited to contribute to the interviews as they wished, and, where appropriate, questions were rephrased to make them more accessible. Although we conducted whole household interviews, for obvious reasons, we did not expect to gain any data from infants who had not yet developed speech and language comprehension. To account for potential power dynamics between parents and children in the interviews, the interviewer explained from the outset that they were interested in hearing from all members of the household and that there may be differing views and experiences within the group that were all relevant to the study. During the interviews, the interviewer was careful to provide space and encouragement to facilitate the children’s contributions, such as asking follow-up questions and prompts to children directly. After each interview, field notes were written, recording such aspects as household and interview dynamics and the researcher’s initial reflections.

**Overview of the topic guide.**

**Consent**

- Motivations for participation and expectations of participating
- The consent process:
  What do you remember?
  Any household or individual concerns
  Any household disagreements
  General feelings about the process
  Would you take part again?
- Information understanding and needs:
  Did the household understand everything they needed to know to participate?
  Areas of difficulty
  Areas where more information is desired
  What information should future participants be told?

**Anonymization and data sharing**

- Feelings about different information collected by SPHERE (Sensor Platform for Healthcare in a Residential Environment)
- Household use of SPHERE Genie
- Expectations about ongoing data use and sharing
- Importance of privacy
- Views about data sharing beyond project researchers

**Feedback on the original SPHERE participant information sheet**

- Views on content and clarity
- Any missing information
- Any extraneous information
- How should information about burdens and risks be presented?

### Data and Analysis

All interviews were double audio-recorded using 256-bit encrypted Olympus digital recorders and an Olympus omnidirectional microphone. Audio recordings were transcribed using a university-approved transcription company. MK checked all transcripts against the recordings for accuracy and removed obvious (direct or indirect) identifying data as part of the process of anonymization. An inductive thematic analysis was undertaken to make sense of the data [21]. This was an iterative process focusing on identifying codes and subsequently developing themes from the data (transcripts) from the bottom-up rather than interrogating the data deductively using predefined codes. Transcripts of interviews were coded and recoded as data collection progressed. The initial coding captured diverse features of the interview texts, and the researcher (MK) developed a coding list with each successive transcript, facilitated by NVivo 10 software. Once all the transcripts were coded, the researcher (MK) used the codes to explore and develop themes to explain the data relevant to the research focus and aims [21]. During this process, members of the research team (MK, RH, JI, and GB) conducted multiple coding across 3 transcripts to check the researcher’s coding interpretations and met to discuss and agree on the development of themes as the analysis progressed, whereby any disagreements were resolved by consensus. The themes were agreed upon by the whole research team and are outlined in the *Results* section.

## Results

### Overview

We derived the following four themes from our data: (1) motivation for participating; (2) transparency, understanding, and consent; (3) privacy, anonymity, and data use; and (4) trust in research. Drawn to participate by their early exposure to the project at a public event or through their existing affiliation with the university, adult participants primarily (and uniformly) described being motivated by an altruistic desire to support research directed toward the public good. Participants described satisfaction with the SPHERE consent process, although they pointed to difficulties in recalling and understanding the information received, the timing and amount of information provision, and the fact that the process seemed, at least initially, to be rather abstract. Participants also reported satisfaction that privacy was assured, taking comfort from the fact that the data were anonymous, not sensitive, and unobtrusively collected, and they felt that any small threat to privacy was compensated by the fact that the data were collected for a worthwhile reason. Participants’ trust in the project and the team was evident, and among the factors relevant to developing and maintaining that trust was the perceived trustworthiness of the research team, the provision of necessary information, the control participants had over participation, and the participants’ positive prior experiences of research involvement.

### Motivations for Participating

Participants described how they came to participate in SPHERE in terms of causal factors, including their early exposure to the project and (for some) their links with the university and their motivations for doing so, which were primarily concerned with benefiting others (other-regarding).

In terms of *causal factors*, households primarily became involved in SPHERE after attending public engagement events or because of existing links to the university. Approximately 71% (5/7) of the households had at least one member who had attended an engagement event. These events were described as informative and “interesting” (P14 from H06). Some participants also noted how attendance had enabled them to contribute to the development of the project, for example, following a “lively debate” about the “level of intrusion we might be prepared to accept” (P01 from H01). Participation in such events was not mentioned by the other 29% (2/7) of households, although those households did refer to an existing affiliation with the university. This was also mentioned by 60% (3/5) of those who attended engagement events.

Participants had a range of *motivations* for becoming involved; however, all had in common an altruistic desire to benefit others, which found expression in participation as they expected SPHERE to generate future health-related benefits. Although the participants were aware that the research would not directly benefit them, some hoped for future personal benefits, as the research might eventually help to address familial circumstances or conditions. Moreover, many anticipated a “[k]ind of public good” (P06 from H02), as they expected the research to benefit others (including the health service at large). Some such benefits were indicated during engagement events:

The second event…it was, kind of like, “This is what it might look like….” In fact, that was one...where for me, the penny dropped about how it might impact on the NHS [National Health Service], given that resources are never going to match demand and here is an electronic solution to a number of monitoring issues.

P01 from H01

Regardless of whether they attended a prestudy event, many of the participants echoed the sentiment that “Most research is useful, eventually, somehow and somewhere” (P13 from H05). Many participants were also motivated by their interest in the research, particularly, as one put it, “the technology side” (P05 from H02). Another participant elaborated as follows:

I thought it sounded interesting, and I think I just thought, “Oh, I’ll just go along and see what it’s all about.” ...I suppose that’s sort of the future use of technology, and supporting people with health conditions and stuff like that seemed interesting and important.

P11 from H04

Other- and self-regarding motivations were dominant and framed as positive reasons for wanting to take part, whereas some participants described a more passive motivation. For example, one (older) child participant felt that the project was interesting and agreed to participate as the other members of the family were willing to do so:

I thought it was interesting, but I just, kind of, went along...because everyone else was.

P03 from H01

No household reported any disagreement about the household’s decision to participate, although this participant implies that the majority view in a given household might prove influential in securing the consent or assent of those who are less motivated. This finding highlights a potential risk with household research that some members may not be actively consenting (or perhaps even assenting) but merely acquiescing and, at worst, doing so as they feel unable to decline to participate. How acceptable this is might depend on the age or autonomy of the individuals in question and the level of potential insult to privacy and autonomy. One way to address this concern is to ensure that all participants freely assent [22] or at least do not dissent when they have a clear opportunity to do so [23]. However, it is not necessarily clear how we should establish assent or dissent in a family environment: consider, for example, a preteen’s mild complaints, a toddler’s tantrums, or an adult with dementia who states that cameras make her feel nervous.

### Transparency, Understanding, and Consent

Whatever their specific motivations for taking part, all households expressed satisfaction with the consent process, including the information received and the opportunity to have their questions answered. However, 5 areas of difficulty and tension also emerged, concerning recall of information, understanding of that information, information overload, timing of information provision, and the possibility that consent might be more theoretical than actual.

First, participants experienced some difficulty in *remembering the* *consent process*, including whether this was a single event or an ongoing process. One household recalled providing consent initially at a pre-event and then again at home before the equipment was installed; however, other households seemed only to recall giving consent at home before or during installation.

Second, various participants revealed *gaps in their understanding*. Some were uncertain about the general nature and remit of the project, with one participant, who had not attended a pre-event, feeling “a bit less clued up...about what the project was” (P06 from H02). Others queried more specific aspects of the project, including what the different pieces of equipment monitored are and why, what the data would look like and how it would be used, and the processes associated with governance and data sharing. Some appeared confused about the practicalities of participation, such as the correct ways to use the equipment and the data that would then be captured, although they were sometimes able to gain reassurance from members of the SPHERE team:

[The technician] came out and explained all the equipment, and how it worked, and showed me some diagrams, and I was aware, at that point, that you were...a silhouette image – so it wouldn’t be taking detailed pictures of you. I don’t think I realised, at that time, that it didn’t take a detailed picture of the background though. I was always a bit conscious that...Because sometimes...the washing up stacks up. I was always thinking “They’ll be able to see all my washing up"...[the researcher] showed me, when she came over, and then I probably felt more comfortable after seeing that. I know [the technician] did show me on a piece of paper, but [the researcher] re-showed me.

P09 from H03

Third, despite these apparent problems with recall and understanding, many households commended the consent process as “thorough” (P02 from H01 and P06 from H02) and “rigorous” (P02 and P04 from H01); however, for at least one household, this also meant that the process was *burdensomely time-consuming*:

So that kind of thing [obtaining informed consent] was only difficult because of my particular pressures that I was dealing with.... So I was like, “Come on, like speed it up.” But obviously I know you’ve got to go through everything because that’s how you make it thorough, so that’s not really a thing anyone can change.

P06 from H02

Finally, participants detected *temporal dimensions* in the consent process; not only did they find it difficult to recall the process many months later, but they also noted how their informational needs had changed over time. Several participants described how they were satisfied that pertinent information had been disclosed before enrollment; however, the length and complexity of the research meant that they had since forgotten that information:

I think, at the time, I felt confident...I felt like everything was covered. Well, they did go into...I’m sure they went into it all. I think it’s just faded in my mind.

P11 from H04

An older participant (P14 from H06) described not only having difficulties recalling information but also preferring not to receive too much written information. Others indicated that their informational needs had changed over the course of the study, with one subsequently becoming interested in how the data generated might be shared with other researchers, observing that “I hadn’t really thought that far down the line with it” (P16 from H07), when initially providing consent. No participants recounted having sought additional information at any point, strongly implying that participants will not necessarily seek further information, particularly about technical and practical matters, after the initial consent interaction.

However, equipped with their knowledge of what participation had involved, some offered suggestions about disclosure to future participants to *make consent more informed and practical*. Many expressed an interest in receiving feedback on the data generated from their household, as well as about how the researchers were using the data collected. However, the latter was more for interest—“There’s no reason for me to know it anyway” (P13 from H05)—and some participants appreciated that the research team would not be able to predict all future uses of the data, as “[i]t’s kind of exploratory research in that way” (P05 from H02). However, many households did indicate that they would have liked more explanation of the practical ramifications of and daily burdens associated with participation rather than those items typically included in information leaflets:

I think, definitely just, I know it’s such a physical thing rather than anything else, but just how much the technology does take up of space around your house, and things...I know it’s so silly, those little things, but I guess it’s just making sure, because I think in your head you’ve got this idea that technology is, well, if you’re doing research it’s going to be really advanced, and it’s all going to be wireless, and everything is going to be like a smart home, type idea, but I don’t think that is what SPHERE was. So, maybe just that, a little bit.

P15 from H07

Practical examples would help participants to better appreciate how the equipment might occupy the home and the routines that would be associated with (for example) using wearable devices; the latter appeared to be the most burdensome (and confusing) aspect of participation. Participants similarly felt that consent might be more authentic and informed if participants could see, in advance, what the data might look like, as otherwise “it’s almost like you’re consenting in theory to something that you don’t quite know what they’re doing, in a way” (P11 from H04).

### Privacy, Anonymization, and Data Use

Participants confirmed that privacy and confidentiality were important to them; however, they expressed few concerns about these, provided that 4 conditions were satisfied: the data collected (and shared) were anonymous, the data were nonsensitive, data collection was unobtrusive; and, consistent with their altruistic motivation, the research (including any follow-on studies) was directed toward the public good.

Privacy was recognized as “[d]efinitely” (P05 from H02) important to participants who were comforted first by SPHERE’s assurance of *anonymity*. The fact that cameras only recorded silhouettes and the data were not being transmitted in real time were considered important privacy safeguards. Participants said they would have been reluctant to participate if more identifiable data were collected: “if we put our names, I don’t think we’d have done it realistically...” (P05 from H02). The provided data were anonymous, and participants were content for it to be used, including by other research teams:

I just think we’re here to, like, provide useful information for other people, but I don’t really mind what they do with it. I’m not really sure what they’re doing with it, but that doesn’t bother me because it’s anonymous.

P03 from H01

Second, participants took comfort from the fact that collected data were *nonsensitive*, even mundane. Ambient sensors and energy- and water-use monitors were viewed as unproblematic—“It’s not like I’m going to be too upset about someone knowing how humid my bathroom is...” (P05 from H02)—and, even if such data were leaked (eg, web-based), anyone viewing said data would “just know...we just walk around the house” (P04 from H01). Some participants were similarly unconcerned about the data collected by the cameras:

I’m not bothered [about camera data] because it is anonymised and, anyway, if someone could find out who it was it, kind of, wouldn’t be very interesting, it wouldn’t matter, and I wouldn’t mind.

P02 from H01

Therefore, the mundanity of the data reassured participants: “We’re not that exciting or perverse...” (P12 from H04). However, the participants did anticipate feeling differently about data collection and sharing, if the data were more sensitive:

I just don’t understand how that would have a negative effect on me. So, as long as it’s not financial or sensitive information, I don’t really mind who knows that about me.

P16 from H07

Third, participants seemed reassured by the *unobtrusive* nature of data collection, which meant that their privacy was not noticeably invaded. SPHERE participants have a mechanism to pause or delete data capture using the SPHERE Genie app; however, it was apparent that use of this was inconsistent across households and some problems with usability were reported. Some households reported that visitors expressed discomfort with the equipment running, and some participants reported being occasionally mindful of the presence of cameras in the home:

When [P10] was little, sometimes I’d be trying to get her in her coat in her pram. It probably looked like I was having a bit of a wrestling match with her. I sometimes thought, “I wonder what, if they see that, they’ll think of that.” Me trying to get her into the pushchair and things, I don’t know what that would look like on there.

P09 from H03

However, many participants soon overcame their initial apprehension and forgot about the monitoring:

I thought that the technology might be more intrusive than it was, so I thought it might get in the way...but it sort of disappeared a lot in your consciousness, isn’t it? Once it was in for a while.

P06 from H02

Awareness of the cameras, for example, “probably lasted for about a week” (P16 from H07), with many participants similarly commenting, “I just forget the equipment is there, and don’t think about it” (P09 from H03) and “I don’t really notice it” (P03 from H01). Indeed, some implied that they would be happy for more data to be collected, so as to provide a fuller picture of their lives beyond their activities in selected rooms. Some participants were also willing to have cameras placed in more private indoor spaces, such as bedrooms and bathrooms (P13 from H05), although the following 2 participants focused instead on outdoor behaviors:

It feels like, then, people should know that I do go out and I do exercise, or I do go out and do a course.

P14 from H06

I just felt like people would get a picture of my life and it’s not how I had it in my head. Does that make any sense? I see myself as quite an active person, quite a social person, but I don’t feel like that would be shown because what they’re actually watching is just me sat down, doing nothing....

P15 from H07

Fourth, participants took comfort in this research being aimed at the *public good*. Despite their evident support for research, this support was limited to research with such aims, such as university-led, health-related research. Furthermore, they tended to feel their data could be shared, provided the recipients had a legitimate interest in the data, as (for example) they were health researchers as opposed to “the News of the World” (P14 from H06) or “organisations like Facebook and WhatsApp who...I think misuse information” (P13 from H05). One participant put it as follows:

I think if they said, “Okay. We’re actually using your information because we want to look at something completely random that’s nothing to do with healthcare,” I don’t think I would feel as comfortable about that, because I don’t feel like, then, that’s what I signed up for.

P15 from H07

Although some were willing to have the data shared beyond the originally envisaged purposes of the project (eg, P03 from H01, quoted earlier), not everyone could recall the possible uses of their data or the processes for handling data access requests.

Participants accepted that the university might create “commercial spin-offs” (P05 from H02) using their data; however, they drew the line at sharing data with commercial companies:

I don’t mind them [the University] making money, but I wouldn’t have felt comfortable with our data about how we live our lives being used to either manipulate us in terms of sales things or really make money for a private company.

P05 from H02

Some participants strongly and consistently expressed their resistance to the data being placed in “some evil company’s hands” (P11 from H04) or being transferred to “some horrible company” (P11 from H04). The corollary of this is that participants appeared to trust SPHERE, the university, and its data-sharing arrangements:

I suppose, potentially, it could go to some unscrupulous researcher who was using it to make vast profits at people’s expense, I don’t know. But, it’s hard to imagine how that could happen, really.

P02 from H01

Ultimately, participants expressed strong support for research that might prove beneficial, which meant they were willing to accept invasions of their privacy:

The level of intrusion you’re prepared to accept probably depends on the level of benefit you see.... If it was more intrusive I might need to be convinced that there was more to be gained...but where we are at the moment makes perfect sense.

P01 from H01

### Trust in Research

Participants exhibited a great deal of trust in the project and the team, which underpinned their perceptions of the adequacy of the arrangements around informed consent, privacy, and data use. Four factors appeared relevant to developing and maintaining their trust: being a trustworthy project and team, transparently providing participants with necessary information, allowing participants control over their participation, and the participants having prior positive research participation experiences.

First, participants felt that the project and team were *trustworthy*. How, exactly, the determination of trustworthiness was made is unclear; however, it seemed connected to the research team meeting the participants’ expectations, their personal experiences with the people undertaking (and overseeing) the research, and the open ethical standards in operation. As we have seen, all 7 households were altruistically motivated, and their trust in the research and the research team that SPHERE was designed to serve the public good (even in the absence of a precise understanding of what that good would or might be) outweighed any concerns they might have had about privacy:

The fact that it was public money that was funding it and all those sort of things was quite reassuring from that point of view, that they weren’t going to be just selling the data on or that their priority was around public good rather than being around monetising the data.

P05 from H02

The involvement of a university rather than a commercial enterprise appeared to instill trust:

I know it sounds crazy that it’s a university, because they are really businesses as well, but they have a set of ethics, they have a set of rules that they’ve got to go by. They’ve got to keep people’s information secure. There has got to be someone that agrees to an industrial company coming in and using that information. So, for me, I guess that’s why I’m not [concerned], because I have a bit more trust in that. It’s research that has come from a research place, I guess.

P15 from H07

Some participants were affiliated with the university, which inclined them to take *a leap of faith* and trust in the project and its team:

I still feel a bit sort of vague about some aspects of it [the research]. But it felt like, I mean [I’m associated with the University], so I kind of feel like there’s a bit of a trust.... I sort of felt like, nice, the sort of researchers at the [University]. So, they must be alright, which maybe explains why, yes, I’ve just, a bit like a leap of faith, I’ve just gone along with things. Whereas, maybe if it had been done by an external company, or a business...I would’ve probably asked more questions. I think that was quite an important aspect of it....

P11 from H04

Even those not affiliated with the (or a) university reported feeling *reassured* that the research was undertaken and overseen in an academic setting: “I felt safe with what was happening” (P16 from H07). SPHERE was judged “to be a very, very ethical operation all the way through” (P01 from H01) that merited trust, whereas, as we have seen, participants were more suspicious of commercial companies:

The SPHERE project, I think, the information collected wouldn’t be misused in any way. But I don’t feel I would have the same confidence about an organisation like Facebook.

P13 from H05

Prior knowledge of or experience with research processes (eg, around privacy, data protection, and data sharing) also inclined participants to trust in SPHERE:

I assume that somebody, someone in SPHERE maybe...has some control over who gets the data, but maybe not. I mean, there must be some mechanism for doing that. I would just assume it’s like with Biobank, I know that the data goes to...people and researchers who have been approved in some way. So, I’ve, kind of, always just assumed that it [SPHERE] would be the same....

P02 from H01

Furthermore, participants recognized their vulnerability, as they were dependent on the project team; however, they felt that the team was mindful of this and deserved their trust:

I think it’s just a question of trust and the objectives and the people who are running it. One gets an impression – impressions are sometimes wrong of course – but you do feel there’s something worthwhile where they would be careful about data.

P13 from H05

You’re assured that it’s anonymous, and to some extent you’re relying on the experts in terms of ethics people and all that to make sure that that’s done properly....

P05 from H02

I just presumed it [data] would be in the right hands and that’s where it would stay....

P16 from H07

Second, participants were inclined to trust as the project *transparently provided what they considered to be appropriate information*. Despite some difficulties with recall, all participants felt sufficiently informed to consent to the participation. The provision of detailed information and the availability of research team members who could answer questions helped to instill trust:

[The researcher] went through loads of forms and talked me through everything and made sure I was very comfortable with everything to do with it [SPHERE].

P06 from H02

A point of contact is good, just so that you’ve got someone to help, isn’t it, and to reassure you....

P16 from H07

Some participants had previous exposure to research or were well acquainted with the research process and research environment and appeared relaxed, even blasé, about providing their consent:

You skim over it [participant information sheet and consent]. You’re so used to doing consent forms for everything, aren’t you? It’s like, “Yes, yes, yes.”

P09 from H03

This is going to sound bad, but...how much do you read it and take it in? Again, it’s that sort of a bit of a leap of faith thing, where like, “Oh, yes, na, na, na, na, na. It’s all the usual stuff,” to a certain extent. It’s good that it’s all there, but it also makes me think...I probably skim read it [participant information sheet] at the time. How much did you take in when you first read it?

P11 from H04

Indeed, some participants appeared to trust the project, seemingly irrespective of the detailed information provided:

I just didn’t think into it too much, I don’t think. I just took it for what it was, and what I was told, and that was okay with me.

P16 from H07

However, others recognized that not everyone would be so trusting:

My father didn’t trust anybody as far as you could throw them, about anything. He wouldn’t have been involved in anything. Like my friend’s husband said, “No, I'm not having strangers coming in and finding out about what we do.”

P14 from H06

Therefore, perceived trustworthiness appeared to play a role in deciding to participate, although participants suspected that less trusting individuals might still come to trust (and participate) in research, provided that they were given detailed information:

If you want people to trust you to be secure, then they need to know that the information is like dot, dot, dot, dot, and nobody could tell who they are...I think it's important for trust, but also for you to get people.... To recruit people for whatever, because people need to have some belief or trust....

P14 from H06

Third, participants trusted SPHERE as they felt they *had control over their participation*. Several of those who had attended SPHERE public engagement events before their participation described this as giving them a sense of control, as they had had an opportunity to contribute to the project during its development:

Part of that might be that we’ve, kind of, grown up with the process...so, actually, to think of that equipment and think, “Actually, I know the process that has got to having that there,” was very, very useful. Not everybody could have that, but for us, what gets recorded is partly influenced by what we said.

P01 from H01

It doesn't bother me [future data use/sharing], because as I say, I was part of the development. I trust the system....

P14 from H06

Participants also valued having control over the data collected, for example, by being able to pause collection or delete data using the *SPHERE Genie* app provided to them on a tablet. One household (H02) was surprised to have this level of influence, as it might adversely affect the volume of data collected. Although not every household used these functions, their value was recognized:

Because I’m not thinking about it [data collection and privacy], I don’t think to use it. It’s nice to think the delete function is there. I do think that’s nice, to think there is that option. I felt more comfortable about the study knowing that was there, but it’s just never been of any relevance to use it. So I’ve not deleted.

P09 from H03

Ultimately, the participants appeared to trust the research and the team. Indeed, all except one of the participants (who felt participation was burdensome) signaled that they would participate again, and 2 households had already agreed to participate for another year:

At the end, I was asked, “Would you be willing to go forward for another year?” “Certainly, yes,” no reason why not.

P13 from H05

Therefore, *positive experiences* of research participation appear to be the fourth factor in maintaining or reinforcing trust (and future participation) in research.

## Discussion

### Principal Findings

For many of these participants, participation was *a question of trust*, and they felt able to take the *leap of faith* and enroll as they trusted the project and its team. The presence of trust seemed to permeate many of the observations that participants offered about their motivations for participating, the consent process, and privacy. Before noting some of the practical implications of these findings, we will first reflect on their ethical dimensions and implications, focusing on the concepts of trust and respect for autonomy.

Trust is a relatively contested concept [24], which is also rather neglected in bioethics [25]. However, Baier [26] has helped to plug the gap, suggesting that trust involves “reliance on others’ competence and willingness to look after, rather than harm, things one cares about.” Reliance alone does not suffice since, as Fritz and Holton [27] note, “Trusting someone involves both a behaviour – a readiness to rely on them – and an attitude,” that is, an assumption that the trusted party is trustworthy, for example, as she is concerned for one’s interests. Nickel [28] further draws out the individual (*internalist*) and institutional (*externalist*) elements of trust, first noting the following:

People’s interest in trust is not merely to have trust, but to have it in the right circumstances and for the right reasons. Normally, this aspect of trust is backed by having a reliable grasp of the interests, functions, and norms that motivate and explain the trusted entity’s behavior.

Internalist accounts of this aspect of trust tend to focus on the *trusting* party: Manson and O’Neill [29], for example, advance an ideal of *intelligent trust*, which emphasizes the characteristics (or virtues) of the truster, who makes sound choices about whom to trust. Externalist accounts look instead to the individual’s social or physical environment and to the *trusted* party, emphasized in such notions as *sound trust* [30].

Our findings chime with these theoretical reflections. The participants noted how some nonparticipating individuals, whether *intelligently*, were not ready to rely on others and, therefore, not prepared to take part in research; however, this was not the attitude of these participants. Rather, our participants evidently were ready *to* trust and, indeed, were trusting *of* the research and the teams involved. They appeared to judge the project and team as worthy of trust and implicitly justified that trust as sound in various ways—usually by appealing to the behaviors of the research team (reflecting an externalist account of trust). The provision of detailed information and assurances about anonymity and data security helped to foster trust; however, participants appeared particularly to base their trust on the fact that the research was, like them, altruistically motivated, a mark of which was that it was led by a university, as opposed to a more self-interested commercial entity.

The research institution in which trust is placed and how it conducts and governs its research appears to matter, and participants were evidently reassured by the ethical goals of the research and the visible ethical standards in operation, which suggests that transparency about ethical standards and ethical practice is important. Time also plays a role here: as Fritz and Holton [27] note, “A trusting relationship will typically be built up over time as we gain evidence that our trust is well placed.” This helps explain why some participants were willing to trust immediately: it was not that they trusted quickly, easily and naively, but rather that they had already built trust in the institution through their existing links and previous experiences. This existing trust enabled them to be unconcerned about areas of uncertainty or, potentially, sanguine about follow-on studies.

The importance of trust in research, which has been noted in other studies [31,32], has ethical implications for future research in smart homes (and, most likely, elsewhere), two of which we note here. First, the institution leading the research should ensure that it is indeed trustworthy. This can be justified not only in ethical but also in prudential terms: our participants imply that any damage to trust will be likely to jeopardize recruitment to future research. Second, attention should be directed to recruitment in any event, as our participants seemed to have trusting attitudes; however, this raises the question of whether less trusting individuals are being (self-) excluded from research. If this is the case, then there is a risk that the research will be biased. As we note below, our participants were all White, (necessarily) self-selecting, and (as they themselves revealed) many were affiliated with the university or at least able to attend pre-events. We wonder, then, what might be missed by failing to engage with and foster trust among other potential participants from more diverse ethnic, cultural, and social backgrounds.

Although trust appeared to be a significant ethical dimension of the findings, participants were also keen to ensure that their autonomy was respected. Respect for autonomy—literally, self-rule—is a dominant concept in bioethics. Amenable to different readings [33], the concept—whether alone or in combination with others—nevertheless underpins or at least connects with such obligations as the need to obtain consent, respect privacy, and maintain confidentiality. Furthermore, it is arguable that trust only has the value and role it does as it is autonomously given in response to trustworthiness.

Turning first to consent, participants were keen to ensure that they had sufficient information before agreeing to participate. They reported being satisfied with the *thorough* information imparted before enrollment and also valued the opportunity to ask questions later to a team member. However, not every participant availed themselves of the opportunity to have their questions answered, and there was evidence of confusion, gaps in knowledge, and desires for further (more practical and less abstract) information to be provided. Problems with participant recall have been recognized in other studies [34]; however, what emerges particularly clearly here is how autonomy is sometimes traded off against or otherwise outweighed by considerations of trust. Autonomy matters, but trust seems to matter more. As such, these participants appeared unconcerned about their inability to recall or at the time fully comprehend study information *just because* they trusted the project and the team, an attitude perhaps best exemplified by the relatively relaxed attitude taken by some participants to study invitations, where participants reported skim reading the information before providing consent.

Some may consider this finding surprising, and it seems at first glance to suggest that participants are less concerned with being in control—with expressing their autonomy—than we might assume. Indeed, this appears to be at odds with the findings of an earlier study that explored the ethical perspectives of (early- and midcareer) smart home researchers [14]. Those participants indicated that they saw the provision of choice to smart home households as offering a solution to the ethical dilemmas, primarily those relating to privacy, which might arise. The current participants certainly valued choice, but they appeared most inclined to trust the researchers, which, contrary to what the researchers themselves indicated, implies that the research team rather than the households is ultimately in control. However, perhaps the respectful and careful attitude of the researchers in their dealings with the participants was central to the trusting relationship that developed.

Trust also appeared to take priority over autonomy when, second, the participants reflected on privacy and confidentiality. Autonomy was again valued in these contexts, with participants pleased to have the opportunity to control the data that were (not) collected and reassured that any such data would be anonymous, mundane, and unobtrusively collected and would (they assumed) only be shared with trustworthy researchers undertaking research in the public interest. However, in practice, not all participants chose to pause data collection or delete the data that had been collected. Furthermore, although anonymity seemed to be important, at least some of the participants signaled that they would be willing to accept greater levels of intrusion and share more data about themselves. These participants appeared willing to trade off both their privacy and their autonomy to support beneficial research, which was linked to their (altruistic) motivations for participating and, ultimately, their trust in the research. The participants’ willingness to have their data used in future health research (but not necessarily in other research) indicates that they may have in mind what Nissenbaum [35] has termed “norms of appropriateness,” which “dictate what information about persons is appropriate, or fitting, to reveal in a particular context.” Sharing beyond such boundaries might result in a privacy violation on Nissenbaum’s account [12,35]. Provided that commercial companies would not receive their data for private gain, participants were also unconcerned that they did not (and perhaps could not) know the future uses to which their data might be put. Sheehan [10], for one, detects no problem with the *broad consent* these participants appear to have in view, as this is still consent and therefore respectful of autonomy. Indeed, according to O’Neill, “[n]either accountability nor informed consent is improved by aiming for high detail and specificity” [36]; rather, she suspects—like our participants—that “any regress of control mechanisms has eventually to end in a decision to place – or refuse – trust” [36].

Relinquishing control to this extent makes participants vulnerable to potential harm; however, participants appeared willing to make themselves vulnerable in this way as they had made the decision to trust. When that decision has been made intelligently, then that trust is an expression of autonomy, and we simply have an obligation to ensure that trust is not breached. Danger may occur when that trust is blind and insufficiently informed to be considered autonomous. However, the appropriate responses appear to be the same.

Therefore, these findings appear to have ethical implications, as far as they indicate that, despite there being a strong emphasis on informing participants and ensuring an autonomous decision in research participation, establishing and maintaining trust may be an essential part of smart home research and that allowing and respecting that trust may be an appropriate way to respect autonomy. If that trust is to be repaid and extended to future studies, researchers should be aware that participants’ agreement to take part in research imposes on the researchers a long-term duty of care toward those participants.

Notwithstanding this general finding that it may be appropriate and acceptable, in future smart home research (and, surely, in many other fields), for respect for autonomy to be viewed and understood through this lens of trust, our findings do also have practical implications for fostering trust. First, the *types of information* imparted in the consent process might be usefully expanded. Beyond the usual information contained in patient information sheets, these participants appeared keen to learn more about the practical ramifications of living with technology. Second, consideration should be given to the *methods of communicating information*. Written information appears useful, but these participants particularly valued (ongoing) in-person communication, including communication before their recruitment during the public engagement events they attended. Third, the thorny question of *feeding findings back to participants* arose here. Some participants sought informational rewards for their participation. However, this might prove challenging for studies of this sort involving whole households, given the need to maintain anonymity and the likely difficulties in disentangling information about multiple participants in a household. This is something that needs to be discussed and, if necessary, negotiated during the household consenting process (with researchers alert to the unequal power dynamics that exist within households, especially those with older children). Fourth, the *promise of anonymity* itself raises questions. These participants appeared to assume that anonymity was guaranteed. We query the viability of an absolute guarantee, and, in the first instance, researchers need to be transparent about this from the outset, given the role that transparency appears to play in fostering trust. However, the presence of trust also implies a long-term duty of ensuring that participant data remain as anonymous as possible. Such a duty may imply that data custodians should work closely with researchers conducting secondary analyses to ensure that participants do not become more identifiable as a result of secondary research. Finally, thought should also be given to *commercial involvement* in the research. Universities appear to be trusted entities, so they may need to exercise caution when collaborating or sharing data with industries. At a minimum, participants will expect to know if or how this is happening; however, the presence of trust may imply longer-term obligations to ensure that the trust is repaid beyond the immediate terms for the consent that has been given.

### Limitations

This study had some limitations. First, although the numbers are adequate for qualitative research with a nonrandomized sample [37], the generalizability or transferability of our findings is necessarily limited, partly because this is qualitative research, but moreover because the sample size was small and the participants were all drawn from a single smart home project, so their views might not be reflective of those involved in other projects. Nevertheless, we consider the exposure of SPHERE participants to prolonged and invasive ubiquitous monitoring to be unique, and thus, research soliciting their insights is justified on this basis. Second, interviewing and conducting inductive thematic analysis are necessarily subjective processes, so other researchers might have derived different themes. However, we achieved thematic saturation and involved the (multidisciplinary) research team in the analysis, so we believe that we have at least given a fair account of the data collected. Third, we could only capture the opinions of a self-selecting sample of participants. The sample only included White participants, and we acknowledge this lack of ethnic diversity as a limitation. To our knowledge, there are no other similar research studies that specifically report on ethnically diverse populations, and unfortunately this lack of diversity appears to be a common problem across other types of research involving human participants [38,39]. Providing opportunities for involvement is a key part of increasing diversity in research [40]; at the time of writing, SPHERE is funding a program of outreach to ethnic minority groups in the city, with the explicit aim that future studies can access the views of more diverse individuals. For SPHERE-CARED, eligible individuals were those who had agreed to participate not only in the SPHERE project but also in further research linked to the project. This means that we could only directly access and represent the views of those willing to participate in the research; those who are unwilling to participate in or even distrustful of the research might well offer different insights. Nevertheless, we note that, in addition to some areas of agreement, our data also captured diverse opinions, including (indirectly) the views of those resistant to participation in research. Finally, although we endeavored to include young children in the interviews (aged 0-5 years), in practice, this proved difficult to achieve as they were too young to follow the interview in full, and when the interviewer posed simpler questions directly to the children, they were shy or unable to articulate themselves; therefore, we were only able to meaningfully use data from older children in the analysis. Furthermore, although every effort was made to create space and opportunity for older children to express themselves during group interviews, it remains possible that existing family power dynamics may have prevented some children (or, indeed, less confident adults) from expressing their opinions [41].

### Conclusions

The SPHERE study offered a distinct opportunity to access the experiences and opinions of participants involved in a smart home research project. Our qualitative study invited willing households to reflect on the practical and ethical dimensions of consent to participation, privacy, anonymization, and data sharing. Although a small study, participants offered insights that might inform future research in this area (and, perhaps, beyond).

Drawn to the project via their existing links to the university or an introductory engagement event, the participants were mainly and uniformly motivated to participate by an altruistic desire to support (health-related) research directed toward the public good. Despite valuing the thorough consent process, the participants revealed certain difficulties with recalling and comprehending the information received, the timing and amount of the information provided, and the fact that the process seemed, at least initially, to be somewhat abstract. Participants also acknowledged the importance of privacy and confidentiality but were reassured by the anonymity and nonsensitive nature of the data collected, its unobtrusive collection, and their belief that they were supporting valuable research, consistent with their altruistic motivation. Notably, participants’ perceptions of informed consent, privacy, and data use, all appeared to be informed by their trust in the project. Among the factors relevant to developing and maintaining their trust were the trustworthiness of the research team, the provision of necessary information, the control participants had over participation, and the participants’ positive prior experiences of involvement in research.

The findings may have practical implications for future research, regarding not only (for example) the types of information researchers should convey and the extent to which anonymity can be assured but also the long-term duty of care owed to participants who had trusted them not only on the basis of this information but also because of their institutional affiliation. Moreover, the propensity to trust according to prior experiences with research or affiliation with research institutions raises an important concern regarding diversity in research participation, whereby researchers should be aware that individuals without these prerequisites may not be so forthcoming in trusting research and offering their participation. There also appear to be important ethical implications: although autonomy matters, trust appears to matter most to these participants. Therefore, researchers should be alert to the need to foster and maintain trust, particularly as failing to do so might have deleterious effects on future research.

## Abbreviations

CARED: Consent and Anonymization: A Review of Ethical Dimensions
NHS: National Health Service
SPHERE: Sensor Platform for Healthcare in a Residential Environment
